# Erratum

**DOI:** 10.4269/ajtmh.17-0677err

**Published:** 2018-07

In Am J Trop Med Hyg 98 (2) by Jones et al (https://doi.org/10.4269/ajtmh.17-0677) there are errors in the footnotes for Tables 1 and 3. Here are the correct versions of these tables.

Corrected Table 1

**Table 1:** Age-specific and age-standardized* weighted prevalence of *Toxoplasma gondii* immunoglobulin G seropositivity among persons aged ≥ 6 years by demographic risk factors overall and among those U.S. born, NHANES 2011–2014

Overall				U.S. born		
Category†	Prevalence	*n*	95% CL	Prevalence	*n*	95% CL
Total unadjusted	11.14	13,507	9.88, 12.51	8.64	10,254	7.40, 10.01
Total age standardized*	10.42	13,507	9.19, 11.76	7.89	10,254	6.66, 9.28
Age group						
6–11	0.91‡	1,757	0.52, 1.48	0.66‡**	1,651	0.29, 1.29
12–19	3.13	2,102	2.00, 4.64	2.44	1,810	1.32, 4.10
20–29	6.85	1,636	5.14, 8.92	4.24	1,271	2.84, 6.05
30–39	9.55	1,685	7.76, 11.59	5.35	1,130	4.01, 6.96
40–49	11.36	1,645	8.86, 14.26	7.46	1,049	4.53, 11.43
50–59	13.27	1,592	10.74, 16.16	9.61	1,076	7.10, 12.65
60–69	17.48	1,586	15.58, 19.51	16.18	1,066	14.07, 18.46
70+	24.58	1,504	21.49, 27.88	23.13	1,201	19.87, 26.65
Gender						
Male	11.25§	6,649	9.69, 12.97	8.45	5,066	6.91, 10.21
Female	9.67	6,858	8.50, 10.95	7.37	5,188	6.21, 8.68
Race and Hispanic origin††						
White, not Hispanic (ref)	8.26	4,971	6.77, 9.95	7.92	4,788	6.39, 9.69
Black, not Hispanic	12.04¶	3,113	10.54, 13.67	9.65	2,834	8.49, 10.90
Mexican American	13.88¶	2,010	11.17, 16.98	6.45	1,196	4.34, 9.16
All Hispanic	19.99#	3,337	16.68, 23.65	5.92	1,767	4.27, 7.97
Asian, non-Hispanic	8.81	1,580	6.97, 10.95	6.75**	432	2.89, 13.05
Place of birth						
Non-United States	22.52#	3,248	19.83, 25.39	NA	NA	NA
United States (ref)	7.89	10,254	6.66, 9.28	7.89	10,254	6.66, 9.28
Poverty index						
Below	16.14#	3,372	14.24, 18.19	11.61¶	2,546	8.85, 14.87
At or above (ref)	9.27	9,144	8.18, 10.46	7.42	7,099	6.39, 8.57
Education level						
< High School	17.93#	3,156	15.69, 20.35	11.41¶	2,026	8.33, 15.13
High School	11.86#	2,910	10.18, 13.71	9.80#	2,346	8.17, 11.63
> High School (ref)	8.07	7,325	7.04, 9.20	6.62§	5,781	5.64, 7.71
Crowding Index (persons per room)						
≥ 1	15.85#	3,124	13.44, 18.49	7.58	2,003	5.06, 10.84
0.5–0.99	11.20#	5,807	9.83, 12.68	8.71§	4,399	7.42, 10.14
< 0.5 (ref)	8.00	4,472	6.70, 9.46	6.98	3,778	5.61, 8.57

CL = confidence limits; NA = not applicable; NHANES = National Health and Nutrition Examination Survey; Ref = reference group.

* All categories except age groups are age-standardized to the 2000 projected U.S. census population using the following age groups: 6–11, 12–19, 20–29, 30–39, 40–49, 50–59, 60–69, and 70 years and above.

† Columns for categories may not add to totals because of item nonresponse.

‡ *P* < 0.001 for linear test for trend among age groups.

§ *P* < 0.05, ¶ *P* < 0.01, # *P* < 0.001 for comparison of subgroup to reference group for each cofactor.

** Relative standard error of the estimate > 30% and estimate may be considered unstable.

†† Total sample sizes for the reported race and Hispanic origin subgroups do not sum to the population total because population total includes those that did not self-select into any of the listed race and Hispanic origin groups or reported as multiracial.

Corrected Table 3

**Table 3:** Age-specific and age-standardized* weighted prevalence of those both *Toxoplasma gondii* immunoglobulin (Ig) M positive and IgG positive among persons aged ≥ 6 years by demographic risk factors, NHANES 2011–2014

Category†	Prevalence	*n*	Lower 95% exact CL	Upper 95% exact CL
Total unadjusted	0.71	13,194	0.54	0.92
Total age standardized*	0.67	13,194	0.51	0.86
Age group				
6–19	0.28‡¶	3,814	0.06	0.82
20–39	0.48	3,072	0.29	0.75
40–59	0.88	3,219	0.49	1.45
60+	1.13	3,089	0.79	1.56
Gender				
Male (ref)	0.68	6,648	0.45	1.00
Female	0.66	6,546	0.47	0.89
Race and Hispanic origin#				
White, non-Hispanic (ref)	0.68	4,854	0.47	0.94
Black, non-Hispanic	0.42	3,039	0.21	0.74
Mexican American	0.22§¶	1,962	0.03	0.74
All Hispanic	0.63¶	3,266	0.31	1.15
Asian	1.28§	1,549	0.76	2.02
Place of birth				
Non-United States	1.16§	3,167	0.78	1.66
United States (ref)	0.58	10,022	0.41	0.80
Poverty index				
Below	0.82	3,268	0.40	1.49
At or above (ref)	0.64	8,955	0.48	0.85
Education level				
< High School	0.91	3,087	0.52	1.47
High School	0.76	2,843	0.40	1.29
> High School (ref)	0.59	7,151	0.36	0.92
Persons per room				
≥ 1	0.55¶	3,021	0.23	1.09
0.5–0.99	1.10§	5,636	0.76	1.54
< 0.5 (ref)	0.46	4,437	0.25	0.78

CL = confidence limits; NHANES = National Health and Nutrition Examination Survey; Ref = reference group.

* All categories except age groups are age standardized to the 2000 U.S. census population using the following age groups: 6–19, 20–39, 40–59, and 60 years and above.

† Columns for categories may not add to totals because of item nonresponse.

‡ *P* < 0.001 for linear test for trend among age groups.

§ *P* < 0.05 for comparison of subgroup to reference group for each cofactor.

¶ Estimate based on < 10 positive sample persons or relative standard error (RSE) > 30% (eight positives and RSE > 50% for those aged 6–19 years, three positives and RSE > 60% for Mexican Americans, RSE > 30% for All Hispanics, and RSE > 30% for households ≥ 1 persons per room) so estimate may be unstable. Results should be interpreted with caution.

# Total sample sizes for the reported race and Hispanic origin subgroups do not sum to the population total because population total includes those who did not self-select into any of the listed race and Hispanic origin groups or reported as multiracial.

The journal regrets the error.

